# Comparison of Optical Quality and Intraocular Scattering after Posterior Chamber Phakic Intraocular Lens with and without a Central Hole (Hole ICL and Conventional ICL) Implantation Using the Double-Pass Instrument

**DOI:** 10.1371/journal.pone.0066846

**Published:** 2013-06-25

**Authors:** Kazutaka Kamiya, Kimiya Shimizu, Aya Saito, Akihito Igarashi, Hidenaga Kobashi

**Affiliations:** Department of Ophthalmology, University of Kitasato School of Medicine, Kanagawa, Japan; Washington University, United States of America

## Abstract

**Purpose:**

To objectively compare the optical quality and the intraocular scattering after implantation of the posterior chamber phakic implantable collamer lens (Visian ICL™, STAAR Surgical) with and without a central artificial hole for moderate to high ametropia.

**Methods:**

This retrospective study comprised 28 eyes of 28 consecutive patients undergoing Hole ICL implantation (mean age ± standard deviation, 30.3±5.8 years), and 24 age-matched eyes of 24 patients undergoing conventional ICL implantation (age, 30.4±6.1 years). We quantitatively assessed the postoperative values of MTF cutoff frequency, Strehl ratio, objective scattering index (OSI), and OQAS values (OVs), using an Optical Quality Analysis System™. We compared these postoperative variables between the two groups.

**Results:**

The mean MTF cutoff frequency, Strehl ratio, OSI, OV100%, OV 20%, and OV9%, were 26.21±8.32 cycles/degree, 0.16±0.04, 1.16±0.57, 0.87±0.28, 0.80±0.35, and 0.80±0.33, respectively, 3 months after Hole ICL implantation. We found no significant differences in the MTF cutoff frequency (Mann Whitney U test, p = 0.59), the Strehl ratio (p = 0.82), the OSI (p = 0.63), or the OVs at contrasts of 100% (p = 0.58), 20% (p = 0.40), and 9% (p = 0.87), between the two groups.

**Conclusions:**

Both Hole ICL and conventional ICL implantation provides an excellent optical performance including intraocular scattering. Newly developed Hole ICL implantation appears to be essentially equivalent in the optical quality variables to conventional ICL implantation, suggesting that the presence of the central artificial hole does not significantly affect the optical quality and the intraocular scattering after surgery.

## Introduction

The Visian Implantable Collamer Lens (ICL™, STAAR Surgical, Nidau, Switzerland), a posterior chamber phakic intraocular lens (IOL) has been reported to be beneficial for the correction of moderate to high ametropia.[1]–[11] In addition, this surgical procedure is largely reversible, and, unlike laser in situ keratomileusis (LASIK), allows the lens to be exchanged, even when unexpected refractive changes occur after surgery. Recently, toric ICL has also been demonstrated to perform well for the correction of high myopic astigmatism.[12]–[17] However, in order to prevent the occurrence of the pupillary block, this surgical technique unavoidably requires preoperative laser iridotomy, which frequently involves some pain, especially in younger subjects, or intraoperative peripheral iridotomy, which is sometimes accompanied with iris hemorrhage, causing difficulties in the surgical procedure. Moreover, there are ongoing concerns about the possible risk of cataract formation, presumably resulting from direct physical contact between the ICL and the crystalline lens or else from malnutrition due to poor circulation of the aqueous humor. We developed a new ICL with a central artificial hole (Hole ICL) in order to rectify such disadvantages. [18]–[20] From a clinical viewpoint, we have already demonstrated in another studies that Hole ICL implantation was good in all measures of safety, efficacy, predictability, and stability throughout the 6-month follow-up period, [21] and that Hole ICL implantation was equivalent in the induction of higher-order aberrations and contrast sensitivity function to conventional ICL implantation. [22] These results indicate that newly developed Hole ICL implantation holds promise as a next-generation surgical option for the correction of moderate to high myopic eyes. Nevertheless, neither the detailed optical quality nor the intraocular scattering, both of which play an important role in the postoperative visual performance and subsequent patient satisfaction, has been so far quantitatively investigated after this novel surgical approach. Considering that the presence of the central artificial hole may deteriorate optical performance, it is of importance to quantitatively evaluate the characterization of the detailed optical quality of the eye including the intraocular scattering in a clinical setting. The purpose of the current study is to objectively compare these optical quality variables in eyes undergoing Hole ICL implantation with those in age-matched eyes undergoing conventional ICL implantation.

## Materials and Methods

### Study Population

Twenty-eight eyes of 28 consecutive patients (7 men and 21 women) who underwent implantation of the posterior chamber phakic implantable collamer lens with a 0.36-mm central artificial hole (Hole ICL™, STAAR Surgical), and twenty-four eyes of 24 age-matched patients (7 men and 17 women) who underwent implantation of the posterior chamber phakic implantable collamer lens without a central hole (conventional ICL™), for the correction of moderate to high myopia (manifest spherical equivalent -4.00 diopter (D) or more) at Kitasato University Hospital, were included in this retrospective observational study. Using the envelope technique, only one eye per subject was selected randomly for statistical analysis. The sample sizes in this study offered 81% statistical power at the 5% level, in order to detect a 6-cycles/degree difference in modulation transfer function (MTF) cut off frequencies between the two groups, when the standard deviation (SD) of the mean difference was 7.5 cycles/degree, and to detect a 0.40-difference in the objective scattering index (OSI) between two groups, when the SD of the mean difference was 0.50. The patient ages were 30.3±5.8 years (mean age ± standard deviation) in the Hole ICL group, and 30.4±6.1 years in the conventional ICL group. In the Hole ICL and conventional ICL groups, we selected non-toric ICL in 20 eyes (71%) and 18 eyes (75%) with the manifest cylinder of 1.25 D or less, or toric ICL in 8 eyes (29%) and 6 eyes (25%) with that of 1.5 D or more, respectively. Eyes with keratoconus were excluded from the study by using the keratoconus screening test of Placido disk videokeratography (TMS-2, Tomey, Nagoya, Japan). The study was approved by the Institutional Review Board at Kitasato University School of Medicine, and followed the tenets of the Declaration of Helsinki. Written informed consent was obtained from all patients after explanation of the nature and possible consequences of the study.

### Implantable Collamer Lens Power Calculation

ICL power calculation was performed by the manufacturer (STAAR Surgical) using a modified vertex formula. In all eyes, emmetropia was selected as the target refraction to minimize preoperative refractive errors. The size of the ICL was also chosen by the manufacturer on the basis of the horizontal corneal diameter and the anterior chamber depth measured with scanning-slit topography (Orbscan IIz, Bausch & Lomb, Rochester, USA).

### Implantable Collamer Lens Surgical Procedure

For conventional ICL implantation, the patients underwent 2 preoperative peripheral iridotomies with a neodymium-YAG laser. For Hole ICL implantation, the patients did not undergo preoperative or intraoperative peripheral iridotomies. On the day of surgery, the patients were administered dilating and cycloplegic agents. After topical anesthesia, a model V4 ICL (Hole ICL or conventional ICL) was inserted through a 3-mm clear corneal incision with the use of an injector cartridge (STAAR Surgical) after placement of a viscosurgical device (Opegan; Santen, Osaka, Japan) into the anterior chamber. The ICL was placed in the posterior chamber, the viscosurgical device was completely washed out of the anterior chamber with balanced salt solution, and a miotic agent was instilled. All surgeries were uneventful and no intraoperative complication was observed. After surgery, steroidal (0.1% betamethasone; Rinderon; Shionogi, Osaka, Japan) and antibiotic (0.5% levofloxacin; Cravit; Santen, Osaka, Japan) medications were administered topically four times daily for two weeks, the dose being reduced gradually thereafter.

### Optical Quality Measurement

We measured the optical quality parameters of the eye, such as the MTF cutoff frequency, the Strehl ratio, the OSI, and the OQAS values (OVs) (100%, 20%, and 9%), 3 months after surgery, using the Optical Quality Analysis System™ (OQAS, Visiometrics, Terrassa, Spain) for a 4.0-mm pupil. The manifest refractive error of the subjects was fully corrected during these measurements; the spherical error (up to -8.00 D) was automatically corrected by the double-pass system, and the residual spherical error (over -8.00 D) as well as the cylindrical error was corrected with an external lens, because the uncorrected refractive error directly affects the optical outcome of the system. The pupil diameter was provided by this device from an image of an additional video camera that allowed pupil alignment. We confirmed that the pupil diameter was more than 4.0 mm in all eyes. The room illumination was kept low (approximately 25 lux) during testing. The value considered is the cutoff point of the MTF curve on the x-axis; the results are given in cycles per degree, representing the highest spatial frequency at lower contrast. The MTF cutoff in the double-pass system is the frequency at which the MTF reaches a value of 0.01. Because the point spread function (PSF) images recorded by the double-pass instrument can be affected by high-frequency noise, which is inherent in the use of cameras, the frequency for very small MTF values may become unstable, potentially leading to artifacts. To avoid this problem, the device uses an MTF threshold value of 0.01, which corresponds to 1% contrast. Thus, the MTF cutoff frequency in this article refers to the frequency up to which the eye can focus an object on the retina with a significant 1% contrast. The Strehl ratio is an expression of the ratio of the central maximum of the illuminance of the PSF in the aberrated eye to the central maximum that would be found in a corresponding aberration-free system. It is the measure of the fractional drop in the peak of the PSF as a function of the wavefront error. The OSI is an objective evaluation of intraocular scattered light. The index is calculated by evaluating the amount of light outside the double-pass retinal intensity PSF image in relation to the amount of light on the center. In the particular case of the instrument OQAS, the central area selected was a circle of a radius of 1 minute of arc, while the peripheral zone was a ring set between 12 and 20 minutes of arc. [23] The OSI for normal eyes would range around 1, while values over 5 would represent highly scattered systems. The three OVs are normalized values of three spatial frequencies, which correspond to MTF values that describe the optical quality of the eye for three contrast conditions, commonly used in ophthalmic practice: 100% (OV 100%), 20% (OV 20%), and 9% (OV 9%). [24] Specifically, the OV100% is directly related to the MTF cutoff frequency (it is the MTF cutoff frequency divided by 30 cycles/degree) and, therefore, to the patient’s visual acuity, although it is not affected by retinal and neural factors. The OV 20% and OV 9% are computed in the same way from smaller frequencies that are linked to 0.05 and 0.1 MTF values, respectively, which maintain the proportion of contrasts of 20% and 9%.

### Statistical Analysis

All statistical analyses were performed using StatView software version 5.0 (SAS, Cary, USA). The Mann-Whitney U test was used to compare the data between the two groups. The results are expressed as mean ± SD, and a value of p<0.05 was considered statistically significant.

## Results

**Figure 1:**
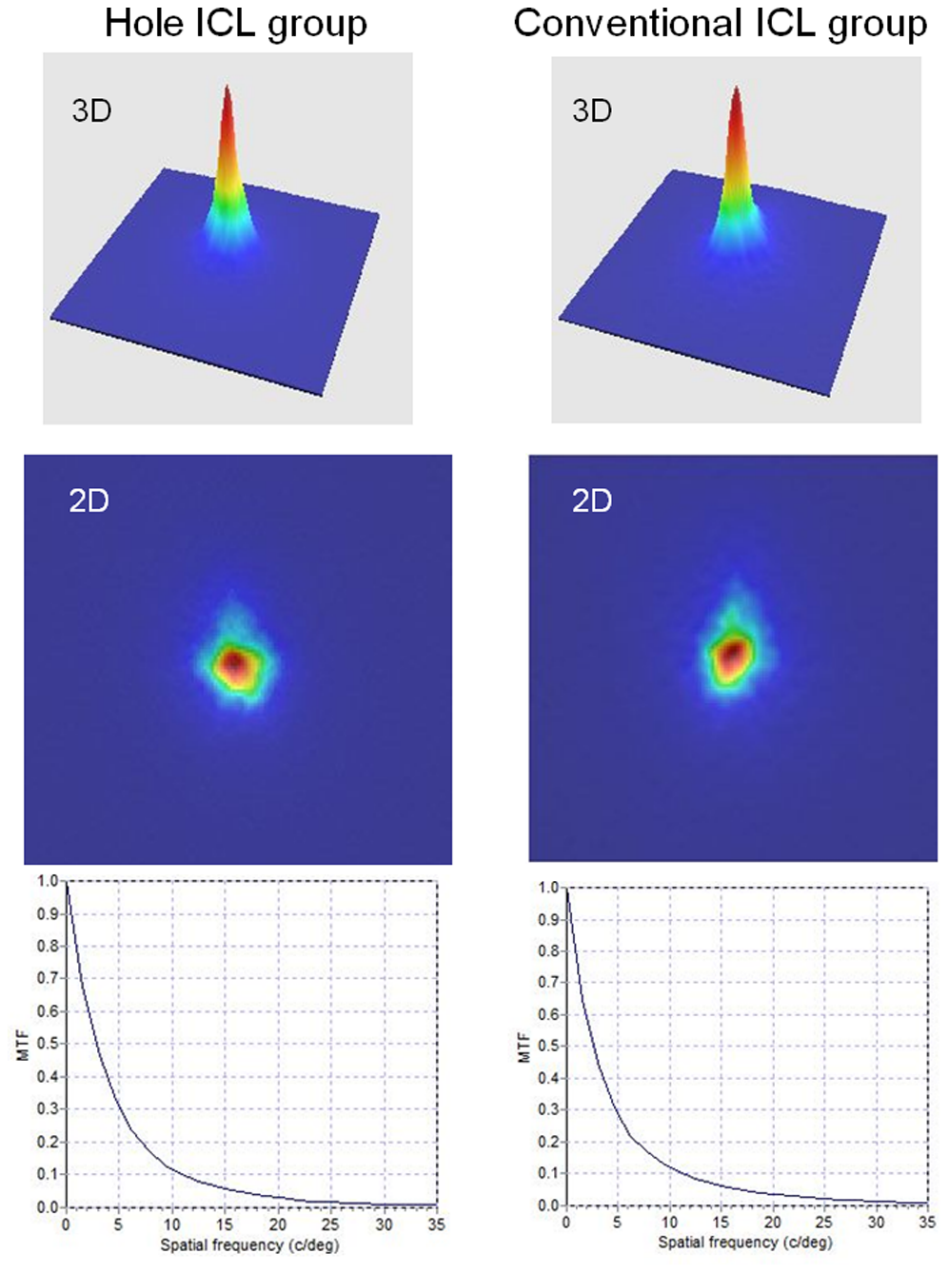
The representative double-pass images of eyes undergoing Hole ICL and conventional ICL implantation.

The demographic data of the study population are shown in Table 1. All surgical procedures were uneventful, and no postoperative complications such as cataract formation, pigment dispersion syndrome, pupillary block, or axis rotation, were seen throughout the 3-month observation period. No eyes were lost during the 3-month follow-up in this series. We found no significant differences in terms of patient age, gender, manifest spherical equivalent, manifest cylinder, logarithm of the minimal angle of resolution (logMAR) uncorrected distance visual acuity, or logMAR corrected distance visual acuity. Figure 1 shows representative examples of the double-pass images of eyes undergoing Hole ICL and conventional ICL implantation. The MTF cutoff frequency, Strehl ratio, and OSI, were 26.21±8.32 cycles/degree, 0.16±0.04, 1.16±0.57, respectively, in the Hole ICL group. The MTF cutoff frequency, the Strehl ratio, and the OSI were 27.58±9.11 cycles/degree, 0.17±0.05, and 1.18±0.53, respectively, in the conventional ICL group. We found no significant differences in the MTF cutoff frequency (Mann Whitney U test, p = 0.59), the Strehl ratio (p = 0.82), or the OSI (p = 0.63), between the two groups (Table 2). The OV100%, OV20%, and OV 9% were 0.87±0.28, 0.80±0.35, and 0.80±0.33, respectively, in the Hole ICL group. The OV100%, OV20%, and OV 9% were 0.92±0.30, 0.84±0.30, and 0.80±0.29, respectively, in the conventional ICL group. We found no significant differences in the OV 100% (p = 0.58), the OV 20% (p = 0.40), or the OV 9% (p = 0.87), between the two groups (Table 2).

**Table 1:** Demographics of eyes undergoing implantable collamer lens (ICL) implantation with and without a central artificial hole.

	Hole ICL group	Conventional ICL group	P value
Age (years)	30.3±5.8 years (95%CI, 19.0 to 41.6 years)	30.4±6.1 years (95%CI, 18.4 to 42.3 years)	0.75
Gender (% female)	36.8%	42.1%	0.82
Preoperative			
Manifest spherical equivalent (D)	−7.17±1.98 D (95%CI, -3.28 to -11.05 D)	−7.24±1.94 D (95%CI, -3.44 to -11.04 D)	0.77
Manifest cylinder (D)	−0.71±0.44 D (95%CI, 0.16 to -1.58 D)	−0.67±0.49 D (95%CI, 0.30 to -1.63 D)	0.65
LogMAR UDVA	1.32±0.18 (95%CI, 0.97 to 1.67)	1.37±0.19 (95%CI, 1.00 to 1.75)	0.17
LogMAR CDVA	−0.22±0.07 (95%CI, -0.09 to -0.35)	−0.21±0.08 (95%CI, -0.06 to -0.36)	0.75
Postoperative (3 months)			
Manifest spherical equivalent (D)	−0.03±0.20 D (95%CI, -0.42 to 0.37 D)	−0.05±0.18 D (95%CI, -0.40 to 0.29 D)	0.88
Manifest cylinder (D)	−0.21±0.35 D (95%CI, -0.89 to 0.46 D)	−0.35±0.35 D (95%CI, -1.05 to 0.34 D)	0.41
LogMAR UDVA	−0.20±0.11 (95%CI, 0.03 to 0.42)	−0.20±0.08 (95%CI, -0.03 to -0.36)	0.17
LogMAR CDVA	−0.26±0.06 (95%CI, -0.14 to -0.38)	−0.27±0.06 (95%CI, -0.16 to -0.38)	0.75

ICL = implantable collamer lens, CI =  confidence interval, D = diopters, LogMAR = logarithm of the minimal angle of resolution, UDVA = uncorrected distance visual acuity, CDVA = corrected distance visual acuity.

**Table 2:** Optical quality parameters in eyes undergoing implantable collamer lens (ICL) implantation with and without a central artificial hole.

	Hole ICL group	Conventional ICL group	P value
MTF cutoff frequency (cpd)	26.21±8.32 cpd (95%CI, 9.91 to 42.52 cpd)	27.58±9.11 cpd (95%CI, 9.73 to 45.42 cpd)	0.59
Strehl ratio	0.16±0.04 (95%CI, 0.08 to 0.25)	0.17±0.05 (95%CI, 0.07 to 0.26)	0.82
OSI	1.16±0.57 (95%CI, 0.40 to 2.48)	1.18±0.53 (95%CI, 0.14 to 2.23)	0.63
OV100%	0.87±0.28 (95%CI, 0.33 to 1.41)	0.92±0.30 (95%CI, 0.32 to 1.51)	0.58
OV20%	0.80±0.35 (95%CI, 0.12 to 1.48)	0.84±0.30 (95%CI, 0.25 to 1.43)	0.40
OV9%	0.80±0.33 (95%CI, 0.15 to 1.45)	0.80±0.29 (95%CI, 0.23 to 1.36)	0.87

ICL = implantable collamer lens, MTF = modulation transfer function, cpd = cycles/degree, CI =  confidence interval, OSI = objective scattering index, OV = optical quality analysis system value.

## Discussion

In the present study, our results showed that the optical quality parameters, namely the MTF cutoff frequency, the Strehl ratio, the OSI, and the OVs at contrasts of 100%, 20%, and 9%, were overall high after Hole ICL implantation as well as conventional ICL implantation, indicating that both ICL implantation provides an excellent optical performance including intraocular scattering. Our results also showed that there were no significant differences in the optical quality parameters between the two groups, implying that the optical quality including the intraocular scattering of eyes undergoing Hole ICL implantation was essentially equivalent to that of eyes undergoing conventional ICL implantation. We previously demonstrated in another studies that Hole ICL implantation was almost equivalent in the induction of higher-order aberrations, contrast sensitivity function, and subjective symptoms such as glare or halos, to conventional ICL implantation, [22] and that the optical quality and the intraocular scattering of eyes undergoing ICL implantation was essentially equivalent to those of healthy eyes. [24] As far as we can ascertain, this is the first published study to objectively assess the detailed optical quality including the intraocular scattering after this novel surgical procedure. Considering that the tilt or decentration of the IOL induces some additional scattering of the eye after phakic IOL implantation, it is clinically important to quantitatively assess this scattering. We assume that the narrow fixated location of the ICL between the iris and the ciliary sulcus may not contribute to any clinically significant tilt or decentration of the lens, [25] thus causing only a small amount of intraocular scattering, as suggested by the low postoperative OSI. However, approximately 40% and 25% of patients, after conventional and Hole ICL implantation, respectively, had difficulty with glare or halo in a clinical setting, although the subjective symptoms were not very severe. Ieong et al stated that night vision symptoms, such as glare or halo, in conventional ICL-implanted eyes were common in the early postoperative period, although most patients expressed a high degree of satisfaction with the outcome of surgery. [26] It is suggested that the presence of a 0.36-mm central artificial hole does not significantly affect overall subjective or objective optical performance for a clinical use. Although edge glare does occur to some extent around the artificial hole, it appears to be clinically acceptable or negligible, since the edge of the myopic ICL around the hole was far thinner than that of conventional IOLs. Actually, Shiratani et al stated that the MTF obtained using the optical simulation software, of an ICL with a 1.0-mm central hole was similar to that of an unperforated ICL. [19] Uozato et al also demonstrated, using the OPAL Vector system, that the differences in MTF between a Hole ICL and a conventional ICL were small and clinically negligible, and that the *in vitro* optical performance of ICLs with a 0.36-mm central hole at various IOL powers fulfills the ISO criterion for MTF, [20] which are in agreement with our clinical findings.

Our limitation to this study is that we did not assess the optical quality parameters of the eyes before surgery. We tried to assess these optical variables before surgery, but this was not very reproducible for quantitative evaluation of these parameters, especially in high myopic (more than -8.00 D) and astigmatic eyes, requiring the combined use of external spherical and cylindrical lenses, possibly because this combination may induce some additional reflection. [24] Accordingly, it still remains unclear whether ICL implantation itself does not induce a change in the optical quality and the intraocular scattering of the eye.

In conclusion, our comparative study showed that the optical quality parameters such as the MTF cutoff frequency, the Strehl ratio, the OSI, or the OVs at contrasts of 100%, 20%, and 9%, were overall high after Hole ICL and conventional ICL implantation, and that these parameters of eyes undergoing Hole ICL implantation were not significantly different from those of eyes undergoing conventional ICL implantation. It is suggested that both ICL surgical procedures provide an excellent optical performance including intraocular scattering, and that the presence of a 0.36-mm central hole does not significantly affect the optical quality including the intraocular scatter of eyes in a clinical setting. We believe that this newly developed Hole ICL implantation is promising as a next-generation surgical option for the correction of moderate to high ametropia, because this approach provides an excellent visual performance almost equivalent to conventional ICL,^19,20,22^ does not require additional peripheral iridotomies, and may reduce the risk of cataract formation.^18,19,21,22^ Further studies with a far greater number of subjects are required in order to confirm these preliminary findings.
